# Skin Bioelectrical Impedance as a Diagnostic Tool in Dermatology: A Literature Review of Current and Emerging Applications

**DOI:** 10.7759/cureus.109589

**Published:** 2026-05-25

**Authors:** Alison C Jimenez Ordoñez, Jesús R García, Diana V Díaz Enríquez, José L Barrera Ariza, Kenia G Fagiani Castillo, Julio C Flores Rodríguez

**Affiliations:** 1 Emergency Department, Hospital San Rafael de Alajuela, San José, CRI; 2 Primary Healthcare, Universidad Autónoma de Guadalajara, Guadalajara, MEX; 3 Dermatology, Consultorio Particular Dra. Diana Díaz, Orlando, USA; 4 Mental Health and Family Medicine, Universidad de Santiago, Santiago de Chile, CHL; 5 Ambulatory Care, Hospital Ambulatorio Multimédica, Guatemala, GTM; 6 Aesthetic and Regenerative Medicine, Clínica Aura, Monterrey, MEX; 7 Aesthetic and Regenerative Medicine, Sociedad Mexicana de Investigación en Medicina Estética (SOMIME), Monterrey, MEX

**Keywords:** bioelectrical impedance analysis, bioelectrical impedance measurement, biomedical applications, clinical dermatology, diagnostic screening tool

## Abstract

Skin bioelectrical impedance and especially electrical impedance spectroscopy (EIS) have become a non-invasive, objective technique with the ability to characterize skin tissue at cellular and structural levels. The literature review uses a narrative synthesis to critically analyze the available evidence on four thematic areas, namely 1) the diagnosis of skin cancer and lesions, 2) the characterization of inflammatory skin diseases, 3) wound healing, and 4) the characterization of skin barrier integrity and hydration. Cross-domain comparison indicates that there are general biophysical effects across all applications, but with varying degrees of clinical validation. The main issues of concern are a critical lack of research standardization procedures, population heterogeneity of study groups, and the deployment of EIS in real-life clinical practices alongside artificial intelligence (AI)-related algorithms. The review ends with a set of research priorities that can be used to promote the translation of an adjunct diagnostic tool toward broader clinical validation across dermatological applications.

## Introduction and background

The skin is the largest human organ, and it is the interface that interacts most with the external environment. It is both a barrier and a sensory organ, an immune interface. Its pathologies range from cosmetically minor to life-threateningly urgent [1]. Among all cancers worldwide, skin cancer is one of the most common types, with melanoma alone accounting for more than 325,000 new cases annually. Despite its high incidence, melanoma's mortality rate is comparatively disproportionate due to its high potential for metastasis, particularly when diagnosed late [2,3]. Inflammatory dermatoses, including atopic dermatitis (AD) and psoriasis, are common, creating a significant, enduring financial strain on healthcare services. For example, AD has a worldwide prevalence of approximately 15-20% among children and 1-3% among adults, demonstrating a growing public health burden [4]. Another significant dermatological issue, chronic wounds, impacts 1%-2% of the population in developed nations, amounting to substantial healthcare expenditures due to lengthy healing processes [5,6]. 

The traditional norm in the diagnosis of these conditions is their clinical visual examination for accurate diagnosis [7,8]. Dermoscopy enhances the diagnostic accuracy of pigmented lesions; however, its diagnostic accuracy drops without adequate training, indicating that sensitivity is operator-dependent and demands specialized training [9,10]. In inflammatory dermatoses, subjective clinical scoring instruments, such as SCORing Atopic Dermatitis (SCORAD), are used to measure disease activity and have been shown to be valid, responsive, and internally consistent; yet, they are criticized for insufficient interobserver reliability and for potentially underestimating actual disease activity by not fully capturing subclinical immunological activity and early barrier dysfunction [11,12]. This dependence on subjective and invasive approaches leaves a quantifiable clinical gap: unnecessary biopsies, delayed diagnoses, and insufficient tracking of therapeutic response.

Bioelectrical impedance is the level of resistance of biological tissues to the flow of low-level alternating electrical current over a series of frequencies [13]. Before describing its clinical applications, it is important to distinguish between the principal measurement modalities used in dermatological research, as these terms are often used imprecisely in the literature: Electrical impedance spectroscopy (EIS) measures impedance across multiple frequencies simultaneously, generating a spectral profile that characterizes tissue at different structural depths. It is the most commonly used modality in dermatological diagnostics, including melanoma detection and barrier assessments. Bioelectrical impedance analysis (BIA) typically applies a single or limited set of frequencies to estimate body or tissue compartment composition, including extracellular and intracellular fluid volumes. Bioimpedance spectroscopy (BIS) is a variant of BIA that uses multiple frequencies to model fluid compartments mathematically, most commonly applied in systemic conditions such as lymphoedema. Electrical impedance tomography (EIT) reconstructs two- or three-dimensional spatial maps of impedance distribution across a tissue region, using multiple electrode arrays. Its dermatological application remains predominantly experimental. These modalities are based on common biophysical foundations, which include pathological alterations of tissue, such as malignant transformation, inflammatory infiltration, and edema, and barrier disruption causes the electrical properties of the cells to change measurably [14]. The three fundamental output parameters include resistance (R), an indicator of extracellular fluid volume and electrolyte distribution; reactance (Xc), an indicator of cell membrane capacitance and cellular mass; and phase angle (PA), an arc tangent of Xc/R, which can be used as an indicator of cellular integrity and vitality [15,16].

The part of the skin epidermis with the largest portion of the total skin impedance is the stratum corneum (SC), or outermost layer of the skin, because its lipid protein matrix is the most densely packed and it is relatively impermeable to ionic currents at low frequencies. Measurement frequency can be increased continuously to higher levels, up to megahertz ranges, so that current penetration through the skin passes through successively lower layers of skin (SC) through the viable epidermis to the dermis, allowing tissue characterization noninvasively and without tissue sampling. As the shape of cells is changed by the disease, membrane integrity, extracellular fluid composition, or lipid packing, the alterations are manifested as a change in impedance that can be measured. The characteristic features of the malignant cells of interest include enlarged nuclei, increased or decreased membrane permeability, and intercellular architecture observable on EIS due to their decreased impedance in comparison with the normal tissue [15-21].

Although theoretically, the biophysical elements are quite convincing in general, a literature review using a systematic approach of the extent and relative impact of EIS applications on the entire scope of dermatological practice is notably lacking. The main focus of the previous literature has been on melanoma detection with the Nevisense (SciBase, Sweden, Stockholm) device, yielding high-quality pivotal trial data that creates a literature that is disease-specific, device-specific, and disproportionate compared to the overall potential of the technology [15]. Its use in inflammatory dermatoses and wound healing, wound care, and barrier functionality has been disseminated throughout bioengineering, dermatology, and wound care literature [16]. Few studies have synthesized them all, and only a handful have compared them with each other in terms of methodological rigor or clinical readiness. Moreover, a number of cross-cutting constraints that have been determined in the impedance literature, such as the derivation of various confounding factors in bioelectrical impedance measurements [17], have not been systematically reviewed in terms of dermatological clinical translation. The growing pace of combining AI with impedance sensing generates more urgency to conduct a review that is analytically based.

There is a need to review current literature to fill the gap discussed. Therefore, the structured narrative literature review synthesizes all evidence on all four main dermatological applications and makes evidence-based recommendations about how many possibilities there are to integrate them into clinical practice and future research.

## Review

Methodology

Review Design

This study is a structured narrative review conducted with a Preferred Reporting Items for Systematic reviews and Meta-Analyses (PRISMA)-adapted systematic search strategy. A narrative design was selected because the included literature is characterized by substantial heterogeneity in study populations, device technologies, frequency ranges, outcome measures, and clinical applications across four dermatological domains. This heterogeneity precludes quantitative synthesis and renders a formal systematic review with a pooled meta-analysis methodologically inappropriate. In accordance with this design, the following PRISMA 2020 elements were intentionally excluded: formal risk-of-bias assessment of individual studies, certainty-of-evidence grading, and statistical synthesis. These exclusions are consistent with published guidance on structured narrative reviews and are acknowledged as limitations of the current evidence synthesis. All other PRISMA 2020 reporting elements, including search strategy transparency, eligibility criteria, study selection documentation, and data extraction, were applied to ensure reproducibility and methodological rigor [22].

Search Strategy

Three electronic databases, such as PubMed, Scopus, and the Cochrane Library, were searched from inception to 31 March, 2026. The literature was searched on 3 and 5 April, 2026. The search strings applied to each database are presented in Table 1. Search terms encompassed all principal modalities of skin bioelectrical impedance measurement, including electrical impedance spectroscopy (EIS), bioelectrical impedance analysis (BIA), bioimpedance spectroscopy (BIS), and electrical impedance tomography (EIT), combined with dermatological condition and outcome terms. No language, date, or publication-type restrictions were applied during the initial search. Manual screening of reference lists of included studies and relevant systematic reviews was subsequently performed to identify additional eligible studies not captured by the electronic search. Gray literature sources and trial registries were not searched, which represents a limitation of this review's search strategy (Table 1).

**Table 1:** Search strings used on each database ti - title; ab/ABS - abstract; kw/KEY - keywords

Databases	Search String
PubMed	("electrical impedance spectroscopy" OR "bioelectrical impedance" OR "bioimpedance" OR "skin impedance" OR "EIS" OR "BIA") AND ("skin" OR "dermatology" OR "dermatological" OR "cutaneous" OR "stratum corneum" OR "epidermis") AND ("diagnosis" OR "diagnostic" OR "monitoring" OR "wound healing" OR "skin cancer" OR "melanoma" OR "dermatitis" OR "psoriasis" OR "skin barrier" OR "hydration") Filters: Full Text, Associated Data.
Scopus	(TITLE-ABS-KEY ("electrical impedance spectroscopy" OR "bioelectrical impedance" OR "bioimpedance" OR "skin impedance" OR "EIS" OR "BIA") AND TITLE-ABS-KEY ("skin" OR "dermatology" OR "dermatological" OR "cutaneous" OR "stratum corneum" OR "epidermis") AND TITLE-ABS-KEY ("diagnosis" OR "diagnostic" OR "monitoring" OR "wound healing" OR "skin cancer" OR "melanoma" OR "dermatitis" OR "psoriasis" OR "skin barrier" OR "hydration").
Cochrane Library	("electrical impedance spectroscopy" OR "bioelectrical impedance" OR "bioimpedance" OR "skin impedance" OR "EIS" OR "BIA"):ti,ab,kw AND ("skin" OR "dermatology" OR "dermatological" OR "cutaneous" OR "stratum corneum" OR "epidermis"):ti,ab,kw AND ("diagnosis" OR "diagnostic" OR "monitoring" OR "wound healing" OR "skin cancer" OR "melanoma" OR "dermatitis" OR "psoriasis" OR "skin barrier" OR "hydration": ti, ab, kw

Eligibility Criteria

Studies were eligible for inclusion if they reported primary research (randomized controlled trials, cohort studies, cross-sectional studies, case-control studies, or prospective and retrospective comparative studies), systematic reviews, or meta-analyses examining any modality of skin bioelectrical impedance in a dermatological context. Preclinical studies were included where findings were explicitly contextualized in relation to clinical translation. Studies published in English and available in full text were eligible. Studies focused exclusively on whole-body composition assessment without skin-specific impedance findings were excluded. Conference abstracts, case reports, editorials, opinion papers, purely theoretical or engineering modeling studies lacking biological validation, and studies published in non-peer-reviewed journals were also excluded.

Selection Process of Studies

**Figure 1:**
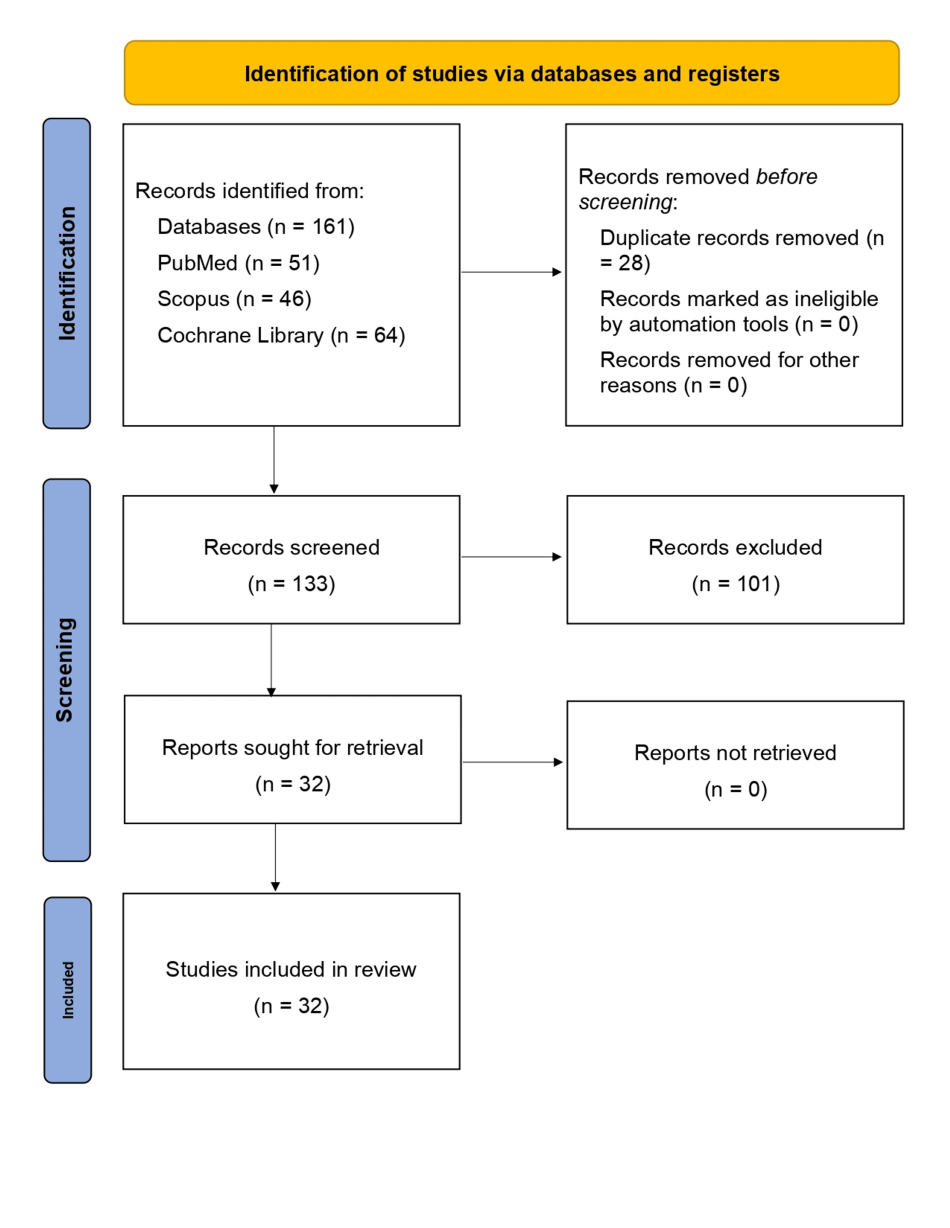
Selection process of studies adapting PRISMA flow chart for narrative literature review PRISMA - Preferred Reporting Items for Systematic reviews and Meta-Analyses

A total of 161 studies were retrieved. After deduplication of 28 studies, 133 were screened to determine their relevance and objective alignment with the literature review by the first two reviewers (ACJO and JRG). However, 101 were excluded due to a lack of relevance. Disagreements between the two reviewers at both screening stages were resolved through structured discussion and consensus. Where consensus could not be reached, a third reviewer (DVDE) was consulted. Studies excluded at the full-text stage were excluded on the basis of the pre-specified eligibility criteria; primary reasons for exclusion included non-dermatological impedance application, non-peer-reviewed publication venue, non-English language, and purely engineering modeling without biological validation. The remaining 32 full-text articles were sought and included in the final literature review synthesis (Figure 1). 

Data Extraction

Data were extracted using a standardized template recording the following variables for each included study: study design, population characteristics, impedance device and measurement parameters (including frequency range and electrode configuration), comparator method, primary and secondary outcomes, and key findings. Data were extracted using a standardized template recording the following variables: study design, population characteristics, impedance device and measurement parameters (including frequency range and electrode configuration), comparator method, primary and secondary outcomes, key quantitative findings, and author-declared conflicts of interest. Extraction was performed independently by two reviewers (ACJO and JLBA) and cross-checked for consistency, with discrepancies resolved through discussion. Formal pilot-testing of the extraction template was not conducted prior to full extraction, which is acknowledged as a methodological limitation.

Narrative Synthesis Approach

The narrative synthesis approach was used. Evidence was grouped into four thematic domains reflecting the principal dermatological applications of EIS: 1) skin cancer and lesion diagnosis, 2) inflammatory dermatoses characterization, 3) wound healing monitoring, and 4) skin barrier and hydration assessment. Within each theme, studies were compared analytically for diagnostic accuracy, comparator validity, and clinical readiness.

The included studies exhibited substantial heterogeneity across multiple dimensions, including impedance device type and manufacturer, electrode configuration (monopolar, bipolar, and tetrapolar), frequency range (from 1 Hz to 2.5 MHz), patient population characteristics, skin condition studied, and outcome measurement approach. This heterogeneity precluded quantitative pooling and is the primary methodological justification for the narrative synthesis approach. Where study findings within a thematic domain appeared to conflict, for example, variable specificity values across melanoma EIS trials or discordant transepidermal water loss (TEWL)-EIS correlations in healthy versus diseased skin, these conflicts are explicitly acknowledged and discussed in the relevant sections. Cross-thematic synthesis examined biophysical principles common across applications, shared methodological limitations, areas of technological convergence (particularly AI integration and wearable platforms), and research gaps requiring prioritization. The cross-thematic comparison was conducted qualitatively, using the three analytical dimensions described above as a consistent evaluative framework across domains.

However, before cross-domain comparison of diagnostic performance metrics, an important methodological caveat must be stated. The sensitivity, specificity, and predictive values reported across the studies included in this review are not directly comparable and should not be interpreted as pooled or equivalent estimates. These values differ as a function of study design (prospective versus retrospective), lesion prevalence in the study population, patient selection criteria (clinically suspicious versus unselected lesions), blinding methodology, reference standard (histopathology versus clinical diagnosis), and impedance device characteristics. All numerical cross-study observations in this discussion are therefore descriptive patterns rather than statistically derived comparative conclusions. Where performance differences between studies are discussed, the relevant methodological differences are explicitly noted.

Evidence synthesis

Theme One: Skin Cancer and Lesion Diagnosis

Melanoma detection - critical evidence: The most extensively developed clinical application of skin EIS is the non-invasive detection of melanoma, which utilizes alternating current at 35 logarithmically distributed frequencies ranging between 1 kHz and 2.5 MHz, offering four penetration depths. The device produces a score of 0-10 (Neviscore), where 0-3 indicates a negative predictive value (NPV) of 99%, and 4-10 suggests an increasing malignant probability [18].

The multicenter prospective blinded clinical melanoma EIS trial by Malvehy et al. [18] has provided the critical evidence base, which was carried out in five US and 17 European study sites, recruiting 1951 study subjects with Fitzpatrick skin type 2 or 3, including 2416 lesions, of which 1943 were biopsied. The study found that malignant melanoma had a sensitivity of 97% and an NPV of 98.2, while its specificity was 34.4%. Sensitivity of non-melanoma skin cancer (NMSC) was 100%. The positive predictive value (PPV) of 21.1% indicates the difficulty of large false-positive rates in a low melanoma prevalence in a clinical population, a trade-off consciously adjusted to high sensitivity to prevent inaccuracies in diagnosis [18].

A previous methodological study by Mohr et al. [19], in a multicenter European prospective trial involving 4790 lesions in 19 centers, confirmed an algorithm-based method with sensitivity values of 98.1% (first algorithm) and 99.4% (second algorithm), with a total value of specificity of 24.5%. Importantly, of melanomas with Breslow thickness less than 1 mm, there are subgroups in which early detection has the most positive influence on the prognosis; the sensitivity was maintained at 97.3%, showing that EIS works accurately in the most clinically impactful diagnostic window. This method is opposed to dermoscopy, where thin melanoma detection is more operator-dependent and less reproducible.

In a 12-center multicenter European blind lesion study by Åberg et al. [20], including 210 lesions and 62 melanomas (38 with 1 mm Breslow thickness), they found significant distinctions between melanoma and benign lesions on the basis of an automated EIS classification algorithm. The data provided evidence of the principle for algorithmic interpretation of EIS without subjective clinician decision-making, which is a vital benefit in standardizing diagnostic thresholds [20].

EIS vs. dermoscopy comparative study: EIS clinical usefulness is best evaluated in comparison to dermoscopy, the non-invasive gold standard for non-invasive pigment lesion assessment [21], in a pilot study by Owji et al. with a survey-based design that assessed biopsy decisions performed by 24 dermatologists, residents, and medical students with either dermoscopic pictures or EIS scores. EIS showed a mean sensitivity of 75% compared to 66% across every assessor (with dermoscopy) (p = 0.008). More importantly, there was the greatest performance gap with a less experienced group; this improvement had a positive effect on residents and medical students. Furthermore, infrequent dermoscopy users experienced greater gains in sensitivity and specificity using EIS than those who used dermoscopy alone. This finding is directionally significant: EIS can operate not only as an adjunct at the level of specialists but also, to a greater extent, as precision across varying levels of clinical experience [21].

Litchman et al. [23] analyzed 267 practicing dermatologists who evaluated 43 lesions and measured this outcome benefit: integrating and executing EIS into clinical decision-making led to an increase in number needed to biopsy (NNB) from 6.3 to 5.3 (p < 0.001), increased sensitivity from 84% to 98% (p < 0.001), and improved specificity from 34% to 44% (p < 0.001). This study resulted in 581 more melanomas being accurately identified through biopsy and 782 fewer needless biopsies in a model population. The improvement was greatest in diagnostically challenging lesions with the fewest ABCD characteristics, precisely in situations where subjective clinical uncertainty is greatest and the objective value added by an adjunct tool is most needed.

Positioning EIS within the broader non-invasive diagnostic landscape: EIS should be understood as one component of an expanding toolkit of non-invasive skin imaging modalities. EIS offers a distinct profile: it does not require optical transparency of the lesion, is unaffected by heavy pigmentation that limits dermoscopic interpretation, and provides biophysical rather than morphological data. Its primary validated advantage over unaided dermoscopy is the reduction in operator dependence, particularly at lower experience levels [21,23]. However, unlike reflectance confocal microscopy (RCM) and optical coherence tomography (OCT), EIS does not generate a visual image of the lesion and thus cannot replace modalities where morphological assessment is clinically required. The optimal clinical positioning of EIS is, therefore, as a complementary, decision-support adjunct-most valuable in the triage of diagnostically uncertain lesions-rather than as a replacement for existing imaging modalities.

Non-melanoma skin cancer - new evidence: EIS evidence for non-melanoma skin cancer (NMSC), in comparison with melanoma, mostly concerning basal cell carcinoma (BCC) and squamous cell carcinoma (SCC), is more focal but encouraging. The pilot study by Saraç et al. [24], including 101 patients and 200 lesions (62 benign, 138 malignant), found a statistically significant difference in impedance between malignant and benign lesions (p < 0.001), and the results indicate that EIS can enhance the performance of NMSC on a high-sensitivity basis. The study encompassed sarcoma, melanocytic naevi, and benign epithelial and dermal tumors, as well as BCC/SCC, thereby expanding the diagnostic capabilities of EIS beyond melanocytic lesions.

A retrospective ecological effectiveness study by Liebich et al. [25] focused on 909 lesions in 481 patients from a privately run dermatology practice that adopted a three-step system, which includes visual examination, dermoscopy, and EIS. The EIS system detected melanomas with 100% accuracy in this real-world environment. Its integration with the clinical workflow decreased NNE (number needed to experience) and has been shown to be clinically effective under normal practice conditions, rather than trial conditions. Nonblinded interpretation of EIS and the retrospective nature of this study, however, limit the strength of conclusions compared to future blinded observations [25]. Table 2 presents the available studies' diagnostic performance.

**Table 2:** Diagnostic performance in lesion detection in various settings *Two separate algorithm iterations reported. †Pre- vs. post-EIS integration values for the same rater cohort. ‡Qualitative descriptor used in original publication; precise values not reported. NR - not reported in original publication; CI - confidence interval; NPV - negative predictive value; PPV - positive predictive value; RCT - randomized controlled trial; NMSC - non-melanoma skin cancer; EIS - electrical impedance spectroscopy Note: Direct numerical comparison across studies is not appropriate due to substantial differences in study design, lesion prevalence, patient selection criteria, blinding methodology, and reference standards. Values should be interpreted within the context of each individual study.

Study	N (lesions)	Design	Sensitivity (melanoma)	CI at 95%	Specificity	CI at 95%	NPV	Setting
Malvehy et al. (2014) [18]	1943	Prospective, blinded RCT	97.0%	95.2-98.3%	34.4%	32.3-36.6%	98.2%	Multicenter (22 sites, EU/US)
Mohr et al. (2013) [19]	1300	Prospective, multicenter	98.1-99.4%*	NR	24.5%	NR	NR	19 European centers
Litchman et al. (2020) [22]	267	Prospective, blinded	84% to 98%‡	NR	34%-44%‡	NR	NR	Hospital settings
Saraç et al., (2020) [23]	200	Prospective pilot	N/R (NMSC focus)	NR	High‡	NR	High‡	University Hospital
Liebich et al. (2022) [24]	909	Trial	100%‡	NR	NR	NR	NR	Hospital setting

Theme Two: Inflammatory Dermatoses

Atopic dermatitis - EIS as a barrier and disease monitoring tool: AD is a disease of the epidermal barrier caused by epidermal barrier dysfunction, including mutations in filaggrin and other structural proteins, which are exacerbated by immune malfunction. Importantly, this barrier failure can be measured through EIS, long before clinical lesions can be observed, establishing EIS as a valuable tool for disease detection, monitoring, and treatment response assessment [26].

A prospective study of AD patients in the hospital by Rinaldi et al. [26] revealed EIS was effective at measuring epithelial barrier integrity, distinguishing between individuals with AD and healthy controls, and characterizing both lesion and non-lesion skin. EIS had a strong inverse correlation with transepidermal water loss (TEWL), the gold standard for barrier assessment, but, crucially, EIS proved more sensitive than TEWL in non-lesion atopic skin from controls [26]. The finding plays a clinically important role: non-lesional atopic skin harbors subclinical barrier disruption; TEWL, already considered one of the most sensitive barrier measures, fails to detect this disruption, whereas EIS does. Values of EIS during hospitalization in lesioned skin, which is associated in a progressive manner with improvement, reduced SCORAD, and lower scores of itch, validating its usefulness as a longitudinal treatment monitor. Furthermore, EIS displayed strong inverse associations with the serum levels of inflammatory biomarkers, such as CCL3, CCL7, CXCL8, CCL13, IRAK1, IRAK4, and FG2, all of which were high at admission and related to barrier disruption. The copy number of filaggrin was associated with EIS on non-lesion skin, indicating that EIS would be useful as a surrogate endpoint, a proxy measure of genetic susceptibility to barrier defects, and a truly novel translational application [26].

Contact dermatitis and irritant reactions: Historically, EIS has been used to characterize irritant and allergic contact reactions of the skin. In a study by Emtestam et al. [27], it was established that changes in skin impedance (frequency-dependent) occurred with both occlusion and exposure to irritants such as sodium lauryl sulfate (SLS), which showed anatomical differences in the skin impedance of five body sites. They went on to draw a comparison between EIS and other non-invasive bioengineering methods (TEWL, blood flow, and skin color), and EIS considered the scoring of visuals in the irritation caused by SLS to be complementary and unique in offering depth-determined data concerning the location of tissue changes. Nyrén et al. [28] examined the possibility of using EIS to help differentiate between allergic and irritant contact dermatitis, a differentiation that is rather difficult clinically and should normally be identified by patch testing over 72-96 hours. The findings indicated that impedance values could differentiate between allergic and irritant reactions, demonstrating the potential of EIS to expedite diagnosis and intervention for contact dermatitis [28].

Psoriasis: Direct clinical evidence for EIS in psoriasis monitoring remains considerably more limited than that available for AD, and the available literature relies substantially on mechanistic inference and barrier model studies rather than condition-specific diagnostic accuracy trials. From a biophysical standpoint, psoriasis is characterized by epidermal hyperproliferation and parakeratosis, resulting in a thickened stratum corneum with abnormal lipid composition. These structural changes are mechanistically expected to increase resistance to current flow, producing higher impedance values compared with normal skin [29]. This direction of change is opposite to that observed in AD, where barrier breakdown reduces impedance-a distinction that positions EIS as a potentially useful tool for differentiating two inflammatory conditions that share overlapping clinical features.

Ahn and Nam (2024) provided direct impedance measurements in psoriasis and cornification models using organotypic artificial skin, demonstrating that EIS could assess barrier function in psoriasis models with sensitivity to structural differences between psoriatic and normal cornification patterns [16]. However, this evidence derives from an in vitro model rather than a clinical population, limiting its direct applicability to diagnostic practice. The systematic review by Iftime et al. (2025) further identified psoriasis-related impedance changes as a documented application of skin bioelectrical impedance while also highlighting the confounding variables that complicate measurement standardization across inflammatory conditions [29].

It should be explicitly acknowledged that prospective clinical trials evaluating EIS diagnostic accuracy or treatment monitoring in psoriasis patients are absent from the current literature. Existing data do not yet support evidence-based clinical recommendations for EIS use in psoriasis. This represents a substantive evidence gap that future adequately powered trials should address, particularly given the availability of effective biologics whose dose adjustment could benefit from objective biomarker monitoring. Table 3 presents the findings of EIS as a barrier and disease-monitoring tool.

**Table 3:** EIS as a barrier and disease monitoring tool for inflammatory dermatoses EIS - electrical impedance spectroscopy; AD - atopic dermatitis; ACD - allergic contact dermatitis; ICD - irritant contact dermatitis; TEWL - transepidermal water loss; SLS - sodium lauryl sulfate

Author (year)	Study design	Condition	EIS application	Key findings	Clinical implications
Rinaldi et al. (2021) [26]	In-vivo study	Atopic dermatitis (AD)	Diagnostic and monitoring tool for epidermal barrier integrity and treatment response	EIS measures intra- and extracellular tissue properties to assess barrier function and monitor healing during therapy.	It is useful for identifying atopy, differentiating lesional/non-lesional skin, predicting AD in infants, and providing an objective evaluation of therapy efficacy.
Emtestan et al. (1997) [27]	Longitudinal experimental study	Contact dermatitis (allergic vs. irritant)	Differentiating allergic (ACD) from irritant (ICD) reactions	EIS indices (MIX, IMIX, and RIX) differ between nickel-induced ACD and SLS-induced ICD despite similar visual appearances.	Suitable for objective differentiation of allergic and irritant natures during patch testing.
Nyrén et al. (2003) [28]	Experimental, comparative study	Irritant contact dermatitis	Comparison with TEWL for SLS-induced irritation	EIS and TEWL both detect irritant reactions, but EIS reflects deeper dermal and epidermal changes.	EIS is as sensitive as or more sensitive than visual scoring in detecting subclinical irritation.
Iftime et al. (2025) [29]	Systematic review	Psoriasis vs. atopic sermatitis	Differential diagnosis based on impedance changes	General impedance levels vary significantly between conditions due to distinct physiological changes.	Potential for EIS to differentiate between clinically similar inflammatory dermatoses, such as psoriasis and AD.

Theme Three: Monitoring of Wound Healing

Biophysical rationale: Wound healing is a continuous process that involves hemostasis, inflammation, proliferation, and remodeling. At each stage, the tissue's electrical properties change distinctly: inflammatory stages involve more infusion of extracellular fluid (ECF) and less Xc; proliferation and cellular migration slowly restore R and Xc towards their original values; and the remodeling phase increases the phase angle towards values related to a healthy state of cell life [30,31]. This is a multi-phase biophysical pathway that creates a unique bioelectrical impedance signature that facilitates therapeutic, non-invasive wound monitoring through continuous measurements, capturing not only wound sizes but also the cellular nature of healing tissue [30,31].

Localized BIA to monitor the wounds: The pioneering description of localized BIA in wounds was brought forth by measurement, with four electrode arrays in the wound margins at 50 kHz with 800 ohms of AC, a current that stimulates neither the tissue of the wound directly nor indirectly [32]. In their series of patients with lower leg wounds, they demonstrated that R, Xc, and phase angle were objective indicators of cellular changes during healing and could alert clinicians to complications before they became clinically apparent. There was a progressive increase in transcutaneous resistance with healing, and impedance identified patients with specific changes in Xc, R, and PA that could reflect altered cellular vitality and fluid balance. The recent study of Antoszewska et al. [33] points to rises in ECF prior to clinical edema appearing, a feature that has already been used in lymphedema staging. These preclinical hints in a wound healing situation translate into susceptibility to wound infection or delayed healing profiles, which can be promptly identified and treated clinically.

Wearable and embedded centralized wound monitoring: The new technology has converted the localized wound BIA into wearable, wireless technology. Song et al. (2023) [34] characterized a wireless, battery-free, and bioresorbable system that allows simultaneous wireless electrotherapy and impedance measurements on the wound site, facilitating real-time, non-invasive wound monitoring and avoiding device removal and wound disturbance. This technology will be a qualitative leap between lab measurements at a single point and instantaneous real-world measurements, a change that, when proven clinically, would effect a significant change in wound care management paradigms. The dual therapeutic-diagnostic potential of bioelectrical wound technologies increased wound healing and monitoring capability in the system [34]. Table 4 presents the role of BIS tools in disease monitoring.

**Table 4:** Wound healing monitoring by BIA BIS - bioimpedance spectroscopy; BIA - bioelectrical impedance analysis; R - resistance; Xc - reactance; PA - phase angle; ECF - extracellular fluid; IL-6 - interleukin 6; IL-10 - interleukin 10; BES - bioresorbable electrotherapy system; NFC - near field communication

Author(s) and Year	Study design	Key findings	Technology/method used
Antoszewska et al. (2024) [33]	Systematic review	We confirmed significant differences in bioimpedance between wounds and healthy skin. Bioimpedance of ulcers consistently increases as the wound surface area decreases. Reactance reflects cell membrane integrity, and phase angle serves as a marker of cellular vitality and fluid balance.	Systematic review of bioimpedance methods, including BIS and BIA; analyzed various electrode configurations (monopolar, bipolar, and tetrapolar) and materials (carbon ink, silver-silver chloride, and copper on polyimide).
Lukaski and Moore (2012) [32]	Case studies	Resistance (R), reactance (Xc), and phase angle (PA) increase during uncomplicated healing, reflecting decreased extracellular fluid (ECF), increased cell mass, and epithelialization. A decrease in these parameters signaled infection before clinical detection.	Localized Bioelectrical Impedance Analysis (BIA): four adhesive surface electrode configurations, phase-sensitive instrument (Quantum IV, RJL Systems), high-frequency (50 kHz), and low-voltage (800 μA AC) current.
Song et al. (2023) [34]	Experimental study (diabetic mouse model)	The wireless, battery-free system provides real-time monitoring via current measurements that correlate with wound dryness and healing progress. Electrostimulation accelerated wound closure by ~30%, enhanced microvascular formation, and modulated inflammation by reducing IL-6 and increasing IL-10.	Bioresorbable, wireless, battery-free electrotherapy system (BES); concentric pair of bioresorbable molybdenum (Mo) serpentine electrodes (15-25 μm thick); NFC-based power harvesting; real-time impedance/current sensing via smartphone interface.

Theme Four: Skin Tone Barrier Health and Hydration Measures

EIS vs. TEWL - comparative values: The clinical and research standard measurement of skin barrier is the TEWL assessment [35]. Nevertheless, it is temperature-sensitive and requires manipulation of temperature and environmental humidity [36], is affected by physical activity [37], and is slow when compared to clinical workflow [38]. Thus, one of the questions is whether EIS could be a reliable, faster, and stronger alternative or complement. The analysis of the EIS by assessing skin barrier function by Huygen et al. [39] has shown that EIS is much less determined by day-to-day routine factors such as washing, exercising, etc., and common environmental changes than TEWL, which yields more consistent values in natural clinical conditions. A high value of negative correlation between EIS and TEWL was established [40]. EIS, which is a relative stabilization tool, is validated as a measure of barrier structure, and its relative stability offers viable benefits for normal clinical practice. Uehara et al. [35] proposed an alternative method for determining TEWL using impedance, which was tested in 25 healthy individuals with intact and tape-stripped (barrier-impaired) skin. This method develops a model based on EIS-derived SC thickness and surface water content and uses these parameters to compute TEWL mathematically. The model had excellent correspondence with the traditional TEWL instrumentation, indicating a future where EIS measurements may be made singly, simultaneously measuring SC hydration, thickness, and barrier functionality, consolidating three separate conventional measurements into one.

SC hydration dynamics: The dynamics of skin hydration during exercise using EIS (in vitro and in vivo) were found to have two different hydration phases: a sudden, rapid decrease in impedance in 5-10 min (filling of electrolytes in and outside the cell subcutaneous spaces) and then a less rapid but more substantial change in impedance indicating macromolecular restructuring of SC barrier elements. This mechanistic detail has direct translational relevance: skin hydration for one hour was considered optimal for transdermal drug delivery, facts that EIS can non-invasively ascertain and verify, aiding studies in dermatopharmacology and cosmetic testing [41].

In-vitro epidermis models/regulatory toxicology: In an article in the Journal of Investigative Dermatology, Brink et al. [42] suggest validating EIS as a rapid, non-invasive technology to measure the barrier function in 3D human epidermal equivalents (HEEs), a gold-standard model of organotypic culture used in clinical and regulatory toxicology and preclinical dermatology. EIS has found two different frequency domain outputs: EISdiff, which is correlated with terminal differentiation of keratinocytes, and EISSC, which is correlated with stratum corneum thickness. Knockout filaggrin CRISPR/Cas9-engineered and claudin-deleted EISdiff directly related the impedance parameters to certain molecules' barrier components [42]. The work provides EIS a chance to be a substitute for animal models in preclinical barrier testing, an important scientific and ethically significant development [43]. Table 5 compares the key findings of studies focusing on skin tone barrier health and hydration measures in TEWL and EIS. 

**Table 5:** Skin tone barrier health and hydration measures comparing TEWL vs. EIS EIS - electrical impedance spectroscopy; TEWL - transepidermal water loss; MIX - impedance index; Z - impedance; 1/Z - inverse of impedance; NE - Nevisense; SCH - skin capacitance; TEER - trans-epithelial electrical resistance; MTT - methylthiazolyldiphenyl-tetrazolium; POST - Plastic Occlusion Stress Test; HP - hydration probe; HEE - human epidermal equivalents Technologies used include various impedance devices: Tewameter (TM Hex, TM 300, TM 210), Nevisense, Corneometer (CM 825), LCR HiTESTER (custom electrodes or setups designed for specific measurement needs)

Author(s) and year	Study design	Key findings	Technology/method used
Uehara and Nakamura (2025) [35]	Clinical study with 25 healthy adult participants comparing intact forearm skin vs. tape-stripped skin barrier function.	There is a strong correlation (R=0.891) between EIS-estimated TEWL and measured TEWL. EIS is a rapid (5 s), stable alternative to conventional TEWL.	The in-house impedance device operates at 500 Hz and 100 kHz, while the Tewameter (TM Hex), confocal laser microscope (VivaScope 1500), and confocal Raman spectrometer are also used.
Huygen et al., (2024) [39]	Clinical study with 31 healthy participants stratified by age (18–29, 30–49, ≥50 years).	EIS is less influenced by daily routine activities (exercise, coffee) than TEWL. No correlation observed between TEWL and EIS (MIX value) in healthy skin.	Nevisense (EIS), Multi Skin Test Center MC 1000 (TEWL index), open-chamber probe.
Kalia et al., (1998) [40]	Longitudinal neonatal monitoring of 10 infants (23 to 32 wk gestational age) in intensive care.	Low frequency impedance (1.6 Hz) is more sensitive to barrier maturation than high frequency parameters. An inverse correlation between 1/Z and TEWL sharpens as a barrier develops (r=0.8).	Low frequency impedance spectroscopy (1 Hz to 1 kHz), Servo Med Evaporimeter EP1 (TEWL).
Brink et al., (2024) [42]	In vitro epidermis model using 3D human epidermal equivalents (HEEs) and CRISPR/Cas9-engineered keratinocytes.	Low frequency (EISdiff) correlates with keratinocyte terminal differentiation. High frequency (EISSC) correlates with stratum corneum thickness. EIS detected therapeutic repair in AD models.	The Locsense Artemis EIS device operates within a frequency range of 10 Hz to 100 kHz and is equipped with a custom gold-plated electrode lid, along with standard HEE cultures.
Morin et al., (2020) [41]	This study examines the comparative hydration dynamics in vivo (human forearm) and in vitro (pig ear).	Hydration occurs in two stages: rapid filling of superficial voids (5-10 min) and subtle structural relaxation over time. There is a good correlation between in vivo and in vitro EIS results.	Nevisense (NE), DermaLab Hydration Probe (HP), and four-electrode Franz cell (4E).
Lotz et al., (2018) [43]	In vitro corneal epithelial model study focused on eye irritation testing (3R method).	Impedance spectroscopy at 1000 Hz (TEER 1000 Hz) is more sensitive than MTT assays and can discriminate all three GHS eye irritation categories by repeated measurements.	The impedance spectrometer LCR HiTESTER 3522-50 is used with a custom measuring chamber for MTT assays.
Jin et al., (2020) [36]	Field study in a Scottish care home with eleven oldest-old (80+) female residents in winter settings.	TEWL measurements were unsuitable for reflecting indoor humidity effects in an inconstant environment. SCH was a better indicator. Median SCH below 50 a.u. indicated dry skin.	The equipment includes the Tewameter TM 300, Corneometer CM 825, Tinytag Data Logger, and Ultrasonic Air Humidifier.
Rosado et al., (2012) [38]	Optimization study of the Plastic Occlusion Stress Test (POST) with 23 female volunteers.	Mathematical modeling of decay curves can reduce TEWL data collection time from 30 min to 10 min. Most water loss occurs in the first minutes post-occlusion.	The Tewameter TM300, Plastic Occlusion Stress Test (POST), and MS Excel Solver are used for bi-compartmental modeling.
Pansang et al., (2010) [37]	Controlled, single-blind skin irritation trial with 30 healthy Thai male subjects using a 3% basil oil microemulsion.	TEWL remained unchanged from baseline in all tested preparations, indicating 3% microemulsion is safe and non-irritating.	We used the Tewameter TM 210, Mexameter MX 18 (erythema index), and a single-application closed patch test.

Discussion

Gradient of Clinical Evidence Across Thematic Domains

Comparison across the four thematic domains reveals a clear gradient in clinical validation and translational readiness. Theme one, melanoma and skin cancer detection, represents the most mature application, supported by multicenter prospective blinded trial evidence [18], regulatory certification across three continents [44] - noting that the lead author declared a conflict of interest as co-founder of the evaluated device, which should be considered when interpreting these findings - and demonstrated practical utility in routine dermatology practice [25]. The pivotal Malvehy et al. trial, encompassing 1943 evaluable lesions across 22 international centers, forms the primary evidence base for this application [18]. Themes two through four, while supported by strong mechanistic rationale and promising preliminary data, lack matched prospective rigor or regulatory validation and represent earlier stages of translational readiness. This gradient reflects both the disproportionate commercial and research investment in melanoma EIS and the greater clinical and financial imperative to reduce unnecessary biopsies, a measurable outcome that has attracted industry-sponsored trial infrastructure not yet replicated for inflammatory or wound healing applications [45].

Sensitivity-Specificity Trade-Off: Implications for Clinical and Policy

Across melanoma EIS trials, consistently high sensitivity (96-99%) is accompanied by comparatively low specificity (24-44%) [18,19,46]. This calibration represents a deliberate design choice: in the context of melanoma, where a missed diagnosis carries life-threatening consequences and an unnecessary biopsy represents a comparatively minor procedural burden, maximizing sensitivity at the expense of specificity is clinically and ethically appropriate. Blundo et al. confirmed that EIS-guided clinical decision-making reduces unnecessary excisions [47], and Litchman et al. demonstrated that EIS integration improves both sensitivity and number needed to biopsy, with the greatest gains observed in diagnostically uncertain lesions [23].

The implications of this trade-off differ substantially in inflammatory dermatoses, where EIS functions as a continuous physiological monitoring tool rather than a binary classifier. In this context, performance is evaluated against reference measures such as TEWL and SCORAD, and sensitivity to treatment-induced change is the relevant metric [26]. Available evidence from Rinaldi et al. suggests EIS is more sensitive than TEWL for detecting subclinical barrier disruption and maintains a strong correlation with clinical disease severity [26]. However, interventional trial data confirming that EIS-guided management improves patient outcomes compared with conventional monitoring are not yet available.

Standardization and Methodological Limitations

There are a number of cross-cutting limitations on the inferences of this review, such as standardization failure. An overview of bioelectrical impedance measurements identified 40 different confounding factors in 10 categories, including electrode type, contact frequency range, anatomical location, medium, body region, age, sex, and ethnicity [29]. Recent physical activity and temperature factors are crucial [29]. Most studies poorly control these variables or report them unmethodically, which invalidates the combination of studies. The absence of a standard measurement protocol that has acquired worldwide recognition (similar to EEMCO guidelines and TEWL) is an important impediment to evidence synthesis and clinical translation. Its absence represents the most important structural barrier to clinical translation across all four thematic domains.

Population heterogeneity is a related and significant concern. The critical trials of melanoma were mainly conducted in Western and North American centers, with patients who were predominantly white-skinned [48]. It is known that the skin impedance differs based on skin phototype and pigmentation [49]. However, these variables are seldom considered in study designs and are referred to as ethnicity [29]. The same concern applies to age; with aging skin, there is a significant change in stratum corneum (SC) hydration, thickness, and lipid structure, which changes impedance patterns [50]. Furthermore, in pediatric dermatology, where atopic dermatitis (AD) prevalence is highest, this population is least represented in EIS validation studies [51].

Artificial Intelligence Integration and Convergence of Emerging Technologies

The interface of EIS in dermatology is at the intersection of impedance sensing with artificial intelligence. In 2022, the FDA reclassified electrical impedance spectrometers as computer-aided devices that provide adjunctive diagnostic details of lesions suspicious for melanoma [52]. This development signals that the regulatory community recognizes AI. EIS convergence constitutes a specialized type of clinical device. Algorithms used in machine learning with multi-frequency, multi-depth impedance spectra, instead of a single Neviscore output, are theoretically able to yield much richer diagnostic information, with the potential to enhance specificity without decreasing sensitivity. This is the most crucial research direction for near-term investigation in this area.

A second frontier is wearable EIS devices. Development of flexible, stretchable electrode materials such as MXenes and conductive hydrogels has created electrodes capable of maintaining performance even in the case of motion [53]. The bioresorbable wireless wound monitoring platform provides proof-of-concept of completely implantable, self-dissolving wound monitoring, a technology that has the potential to change chronic wound management through offering uninterrupted healing information, reducing the need for device removal, and decreasing both the risk and burden of infection and nursing care. The epidermal equivalents of in vitro characterization of EIS are a third frontier: animal models are being replaced or decreased by the use of EIS in preclinical skincare studies and regulatory toxicology, a novel advancement that has a scientific and regulatory component and ethical significance [42].

Research Priorities and Future Recommendations

According to this synthesis, we identify the following research priorities: priority one is uniformity of measurement guidelines. A multi-stakeholder consensus process, by including dermatologists, biomedical engineers, device producers, and regulators, ought to institute minimum reporting standards for studies on skin EIS, similar to the CONSORT or STROBE statements. Some of the variables to be required are: electrode type and spatial resolution; applied frequency region and intensity; skin preparation; anatomical location(s); patient hydration; ambient temperature; and recent topical treatment washout periods.

Priority two is diverse and representative populations. Future prospective trials should be stratified by skin phototype (Fitzpatrick I-VI), age (pediatric-elderly), and ethnicity, including predetermined subgroup analyses. The existing evidence base primarily reflects light-skinned adult populations, indicating a lack of scientific and ethical rationality that must be addressed before making global clinical recommendations [54]. Priority three is trial criteria: longitudinal monitoring. Future research evaluating EIS-guided versus conventional treatment responses in inflammatory dermatoses is a pressing need. An RCT comparing EIS-guided biologic initiation/dose adjustment with conventional management of AD and psoriasis, guided by SCORAD/PASI, could establish the clinical significance of EIS and determine whether it offers any clinical benefit.

Priority four is the integration and algorithm transparency of AI. Artificial intelligence (AI) is a branch of research, and transparency in algorithms should be a required feature of multi-frequency EIS data, externally validated using independent datasets. Similar reporting standards to the TRIPOD guidelines for prediction model reporting should be embraced. Priority five is the assessment of health technology. Cost-effectiveness analyses using new HTA frameworks (QALY, ICER) are needed for EIS in melanoma detection, including real-world data on NNB and biopsy costs. For wound monitoring, the cost-offset potential of early complication detection can and must be quantified using economic models with continuous bioimpedance monitoring. Priority six is trials of non-melanoma and inflammatory conditions. Adequately powered potential applications of EIS include BCC/SCC diagnosis, AD monitoring, and chronic wound healing. Forecasting of the trajectories will be required to cover evidence gaps in themes two to four of this review.

## Conclusions

Skin bioelectrical impedance, and EIS in particular, is a non-invasive, biophysically grounded diagnostic technology with demonstrated clinical efficacy in melanoma detection and emerging evidence across a broad range of dermatological applications. In melanoma detection, EIS achieves a sensitivity of 97-99%; outperforms unaided dermoscopy, particularly at lower experience levels; reduces unnecessary biopsies; and has been validated in international multicenter trials with regulatory approval on three continents. Beyond melanoma, evidence supports EIS as a more sensitive detector of subclinical barrier disruption than TEWL in AD, a cellular-level monitor of wound healing trajectories, and a stable, environmentally robust measure of stratum corneum hydration. These capabilities rest on a mechanistically coherent biophysical foundation. However, the translational potential of EIS across themes two through four remains constrained by a fragmented evidence base, the absence of measurement standardization, population homogeneity in existing trials, and the early clinical readiness of non-melanoma applications. The field now requires the transition from proof-of-concept studies to adequately powered, diverse, multicenter trials with patient-relevant and health-economic outcomes. The convergence of EIS with artificial intelligence, wearable flexible electronics, and in vitro organ model validation signals rapid technological maturation. If the research priorities identified in this review - standardization, population diversity, longitudinal monitoring trials, and health technology assessment - are pursued systematically, skin bioelectrical impedance has the potential to evolve from a specialist-level melanoma adjunct into a foundational, cross-application diagnostic and monitoring technology across dermatological practice. The biological and mechanistic case is established; the clinical evidence base across its full range of applications now awaits confirmation through rigorous future trials.
